# Preventing Soft Tissue Complications in Secondary Aesthetic Breast Surgery Using Indocyanin Green Angiography

**DOI:** 10.1093/asj/sjac261

**Published:** 2022-10-15

**Authors:** Marzia Salgarello, Valentina Pino, Domenico Maria Pagliara, Giuseppe Visconti

**Affiliations:** Dr Salgarello is an associate professor and plastic surgeon, Department of Plastic Surgery, Dipartimento per la Salute della Donna, del Bambino e di Sanità Pubblica—Fondazione Policlinico Universitario “Agostino Gemelli” IRCCS—Università Cattolica del “Sacro Cuore” Rome, Italy; Dr. Pino is a plastic surgeon, Department of Plastic Surgery, Dipartimento per la Salute della Donna, del Bambino e di Sanità Pubblica—Fondazione Policlinico Universitario “Agostino Gemelli” IRCCS—Università Cattolica del “Sacro Cuore” Rome, Italy; Dr Pagliara is a plastic surgeon, Department of Plastic Surgery, Mater Olbia Hospital, Olbia, Italy; Visconti is a plastic surgeon, Department of Plastic Surgery, Dipartimento per la Salute della Donna, del Bambino e di Sanità Pubblica—Fondazione Policlinico Universitario “Agostino Gemelli” IRCCS—Università Cattolica del “Sacro Cuore” Rome, Italy

## Abstract

**Background:**

Secondary cosmetic breast surgery after primary augmentation with implant can be associated with an increased risk of adverse events. Partial/complete nipple-areola complex necrosis is particularly feared. In this preliminary study, the authors propose the utilization of indocyanine green (ICG) angiography to assess the blood supply of breast tissue after implant removal.

**Objectives:**

The main objective was to prevent skin and gland necrosis in revision breast surgery.

**Methods:**

The authors performed a retrospective comparative analysis of 33 patients who underwent secondary breast surgery between 2018 and 2021 by a single surgeon (M.S.). Breast tissue perfusion was assessed in 16 patients by intraoperative ICG angiography at the end of implant removal and possible capsulectomy. Non-stained/non-fluorescent areas were judged to be low perfusion areas and were excised with short scar mastopexy.

**Results:**

In the ICG angiography group, 7 patients (44%) showed an area of poor perfusion along the inferior pole; all of these patients underwent subglandular breast augmentation. Resection of the poor perfusion areas allowed an uneventful postoperative course. In the non­­- ICG angiography group (17 patients), 5 patients experienced vertical-scar dehiscence/necrosis. We found a statistically significant association between the non-ICG angiography group and vertical scar dehiscence/necrosis, and also between vertical scar dehiscence/necrosis and subglandular implant placement (*P* = 0.04).

**Conclusions:**

Safer secondary surgery can be offered to patients undergoing secondary aesthetic breast procedures, especially when the first augmentation surgery is unknown—for example, implant plane, type of pedicle employed, if the implant is large and subglandular, and if capsulectomy is performed.

**Level of Evidence: 4:**

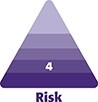

See the Commentary on this article here.

Breast augmentation with implants is the most popular cosmetic surgery, with a reported 354,753 procedures performed in 2021^1^; consequently, secondary breast surgery with request for implant replacement is becoming more and more frequent, usually dictated by implant complications or deterioration of the cosmetic results related to pregnancy/aging.^2–5^ Secondary surgery can be associated with an increased risk of adverse events. However, in literature, there are few data regarding complications and poor results in augmented patients undergoing secondary breast surgery. Many of these can be related to perfusion defects after previous surgery. Teng et al^6^ used 3T magnetic resonance imaging to examine the in vivo perfusion of the nipple-areola complex (NAC) in women with breast implants to know which blood supply prevails and thus spare the areas of the breast with the most robust blood perfusion patterns in future dissections.

Partial or complete NAC necrosis is particularly feared. Impaired scar healing with wound dehiscence is another feared complication. Consequently, maintaining perfusion to the NAC and inferior pole tissues is a critical aspect of secondary breast surgery to avoid skin and gland ischemia and its consequences.

A critical subset of patients is represented by those with a large implant placed subglandularly and a small amount of residual breast tissue. In fact, performing the subglandular pocket requires the sacrifice of tissue perfusion derived from Wuringer septum perforators. This makes the tissues of the inferior breast pole the most peripheral-perfused area of the breast. In addition, the soft tissue of the breast is stretched and thinned by the implant over time, and further surgery may compromise the blood perfusion.

Any secondary surgery that includes capsulectomy can further compromise blood supply to the breast tissue and the NAC. It is not always clear whether partial or total capsule removal is appropriate. In the literature, total capsulectomy is recommended in case of complicated capsular contracture or breast implant illness and at patient request.^7^ Currently, available evidence indicates the use of en bloc capsulectomy in patients with breast implant–associated lymphoma.^8^

In addition, total capsulectomy is easily accomplished with subglandular breast implants but imposes more significant risks when attempted in an adherent capsule along the chest wall in submuscular breast implants.^7^ However, the appropriate surgical approach for each patient requires a significant amount of discernment by the plastic surgeon. The purpose of this study was to propose the utilization of indocyanine green (ICG) angiography to assess blood supply to breast tissue after implant removal to prevent NAC, mammary skin, and gland necrosis in the breast revision surgery.

## Methods

We performed a retrospective comparative analysis of patients who underwent secondary aesthetic breast surgery, between September 2018 and December 2021, by a single surgeon (M.S.) for implant-related issues. We enrolled 33 consecutive patients with previous bilateral breast augmentations (between 1995 and 2015) and capsular contracture, implant rupture, or patient choice for aesthetic improvement. The capsular contracture was assessed employing the 4-degree Baker score,^9^ and the implant rupture was evaluated with ultrasound and magnetic resonance imaging. According to preoperative conditions, patient expectations, and preference for the size of breast implant, we planned implant replacement and mastopexy. Informed consent was widely explained and acquired from all patients. This study conforms to the Declaration of Helsinki ethical principles for medical research. Baseline measures of demographic–anthropometric variables (age, weight, height, BMI) and clinical and surgical factors (smoke, comorbidities, breast incision at the first surgery, implant position [ie, subglandular or submuscular], and size of the removed implant) were collected from preoperative evaluation in the medical records. Breast tissue perfusion was assessed by ICG angiography in 16 patients at the end of partial or total capsulectomy and implant replacement. The near-infrared camera Fluobeam Clinical System (Fluoptics, Grenoble, France) was utilized to acquire fluorescent images of breast perfusion. No local infiltration with adrenaline was employed in order to avoid any interference on the assessment of the breast perfusion. ICG (0.25 mg/kg) in 5 mL of injection water was injected intravenously, and the area enhanced in 90 seconds after injection was assessed.^10^ The unstained/not fluorescent area was judged as a poorly perfused area, its perimeter was marked under the guidance of the ICG angiography, and its resection was performed.

The excess of the skin envelope was managed with short-scar mastopexy, that is, vertical mastopexy or J-scar mastopexy.^11–14^ For the central skin excess, we gathered the periareolar skin with a purse-string stitch (gathering suture) and then marked and opened the neo-areola. At the end, we sutured the skin with the interlocking suture.^15,16^ We recorded data related to postoperative complications at 3, 6, and 12 months of follow-up.

All statistical analyses were performed with IBM SPSS Statistics 20 (Armonk, NY, USA). We compared the data of the ICG angiography group (16 patients) vs the non-ICG angiography group (17 patients). We employed non-parametric tests because there was no normal distribution of values. We carried out the analysis of demographic–anthropometric data and preoperative clinical/surgical factors in the 2 groups. We investigated the presence of statistically significant differences in infection, seroma, NAC dehiscence/necrosis, vertical-scar dehiscence/necrosis in ICG angiography, and non-ICG angiography groups utilizing Fisher's exact test. We also investigated the correlation between total capsulectomy and hypoperfusion in the ICG angiography group. Lastly, the presence of statistically significant differences in the complication rate of the subglandular and submuscular implant position in the 2 groups was analyzed.

## Results

The demographic–anthropometric data of the sample are shown in Table 1. The mean age of the patients in the ICG angiography group was 55.83 ± 9.81 and in the non-ICG angiography group was 51.19 ± 9.23 with a range from 37 to 73 years.

**Table 1.** Demographic and Anthropometric Variables in ICG Angiography and Non-ICG Angiography Groups

Variable	ICG angiography group, n = 16	Non-ICG angiography group, n = 17	*P*
Age, y	55.83 ± 9.81	51.19 ± 9.23	0.21
Weight, kg	61.25 ± 5.51	63.81 ± 6.42	0.29
Height, cm	165.08 ± 6.17	166.57 ± 4.98	0.42
BMI	22.43 ± 0.88	22.94 ± 1.39	0.44

All data in columns are mean ± standard deviation. The *P* values were obtained from the Mann-Whitney U-test. There were no statistically significant differences in age, weight, height, and BMI between the 2 groups. The significance level is 0.05. ICG, indocyanin green.

In the ICG angiography group, 6 patient were smokers and 2 reported arterial hypertension. In non-ICG angiography group, 5 patients were smokers and 2 reported arterial hypertension. No other comorbidities were detected in both groups. As underlined by Fisher's exact test, we found no statistically significant association between ICG angiography group and smoke (*P* = 0.67) or arterial hypertension (*P* = 0.45).

The patients underwent previous bilateral breast augmentation through inferior periareolar incision (9 patients in ICG angiography group, 7 patients in non-ICG angiography group), inframammary incision (3 patients in ICG angiography group, 5 patients in non-ICG angiography group), inverted-T incision (3 patients in ICG angiography group, 2 patients in non-ICG angiography group), or short-scar mastopexy incision (1 patients in ICG angiography group, 3 patients in non-ICG angiography group). The implant position was subglandular in 8 patients of the ICG angiography group and 9 patients of the non-ICG angiography group, and submuscular in 8 patients of both the ICG angiography and non-ICG angiography groups. The indication for secondary surgery was capsular contracture in 12 patients of both the ICG angiography and non-ICG angiography groups, implant rupture in 2 patients of the ICG angiography group vs 2 patients of the non-ICG angiography group, and patient choice in 2 patients of the ICG angiography group vs 3 patients of the non-ICG angiography group.

All patients underwent secondary breast surgery employing a short-scar mastopexy technique, that is, 12 patients underwent vertical-scar mastopexy and 21 patients underwent J-scar mastopexy. The mean size of implant explanted was 315.94 cc (±81.42 cc standard deviation [SD]) in the ICG angiography group and 303.53 cc (±68.91 cc SD) in the non-ICG angiography group. No statistically significant differences were found in distribution of the mean size of implant explanted with the Mann-Whitney U-test (*P* = 0.63).

The mean size of the new breast implant placed in secondary surgery was 340 cc (±75.01 cc SD) in the ICG angiography group and 337.94 cc (±58.71 cc SD) in the NON-ICG angiography group. No statistically significant differences were found in distribution of the mean size of the new implant with the Mann-Whitney U-test (*P* = 0.81).

**Figure 1.**
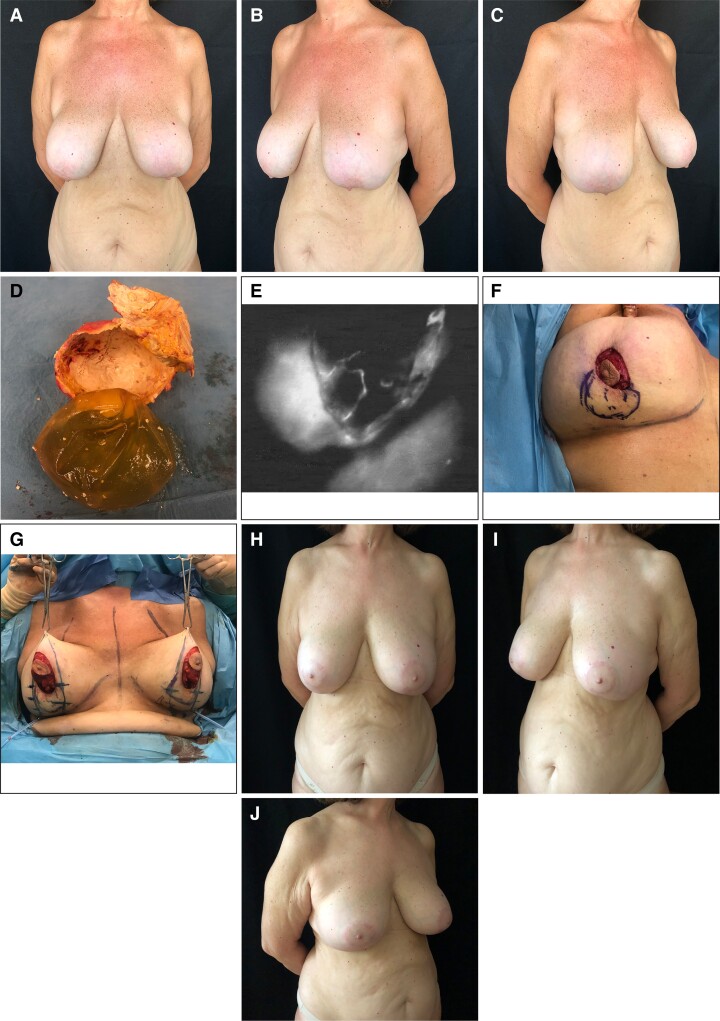
This 62-year-old female patient presented with bilateral breast pain, breast shape deformity, and bilateral Baker 4 capsular contracture. She underwent subglandular breast augmentation 40 years previously. Preoperative (A) frontal view and (B, C) lateral views. (D) Calcified capsules were found after bilateral total capsulectomy and implant removal through the inferior periareolar scar of the previous surgery. (E) Breast tissue perfusion was assessed with the indocyanin green angiography. (F) An unstained indocyanin green area was detected bilaterally at the intersection of the lower quadrants and marked. (G) Anatomical 295-cc implants were inserted in the subglandular pocket, and J-scar mastopexy was performed enclosing the unstained breast tissue in the vertical excision area. Postoperative (H) frontal and (I, J) lateral views at 12-month follow-up.

Intraoperative ICG angiography identified a poorly perfused breast area in the center of the lower quadrants in 7 of 16 patients (44%); all of these patients underwent subglandular breast augmentation. In all these cases, the hypoperfused area was included in the short-scar mastopexy markings, and the consequential trimming was carried out (Figure 1). In 2 out of 7 cases of poorly perfused breast, the hypovascularization involved the center of the lower quadrants and extended under the NAC; then the hypovascularized tissue was trimmed with the short-scar mastopexy without further scarring.

Total capsulectomy was performed in 5 of 16 patients in the ICG angiography group, and poor breast tissue perfusion was found in 4 patients, all of whom underwent trimming.

One case of postoperative infection occurred in the non-ICG angiography group, which was solved with intravenous antibiotics. No case of seroma was found in either group. As underlined by Fisher's exact test, there was no association between groups and infection (*P* = 0.51).

We found no statistically significant differences in the 2 groups in the correlation between the type of mastopexy and risk of dehiscence; the dehiscence/necrosis rate was comparable between the 2 types of short-scar mastopexy, that is, vertical mastopexy or mastopexy with scar J.

**Figure 2.**
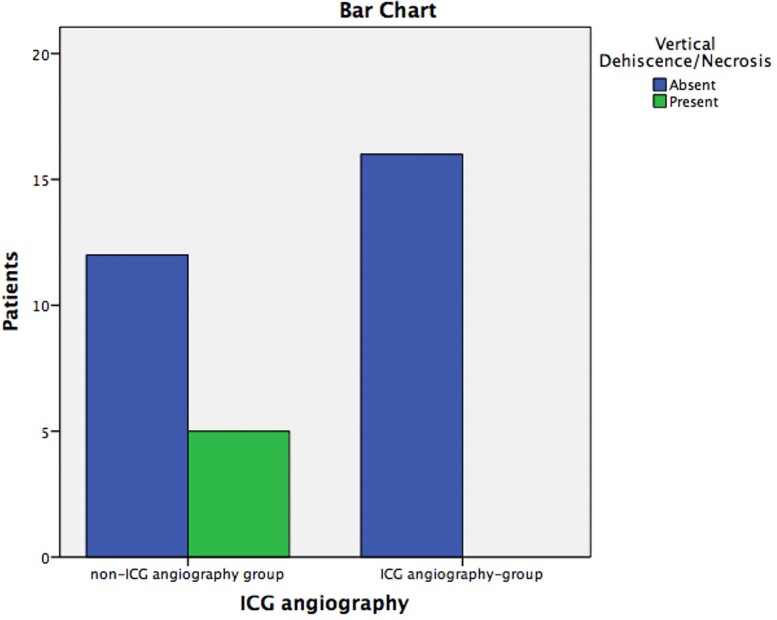
Clustered bar charts of vertical-scar dehiscence/necrosis. In the non-indocyanin green (ICG) angiography group, 5 patients showed vertical-scar dehiscence/necrosis vs 0 patients in the indocyanin green angiography group.

In the non-ICG angiography group (17 patients), 5 patients experienced vertical-scar dehiscence/necrosis (Table 2). As underlined by Fisher's exact test, we found a statistically significant association between the non-ICG angiography group and vertical-scar dehiscence/necrosis (Figure 2). We detected a statistically significant association between vertical-scar dehiscence/necrosis and subglandular implant placement (*P* = 0.04).

**Table 2.** Frequencies and Fisher's Exact Test for Dehiscence/Necrosis in ICG Angiography and Non-ICG Angiography Groups

Complication	ICG angiography (n = 16)	Non ICG angiography (n = 17)	*P*
NAC dehiscence/necrosis	0	0	0
Vertical-scar dehiscence/necrosis	0	5 (29%)	0.04

In the non-ICG angiography group (17 patients), 5 patients experienced vertical-scar dehiscence/necrosis. The significance level is 0.05. ICG, indocyanin green; NAC, nipple-areola complex.

**Figure 3.**
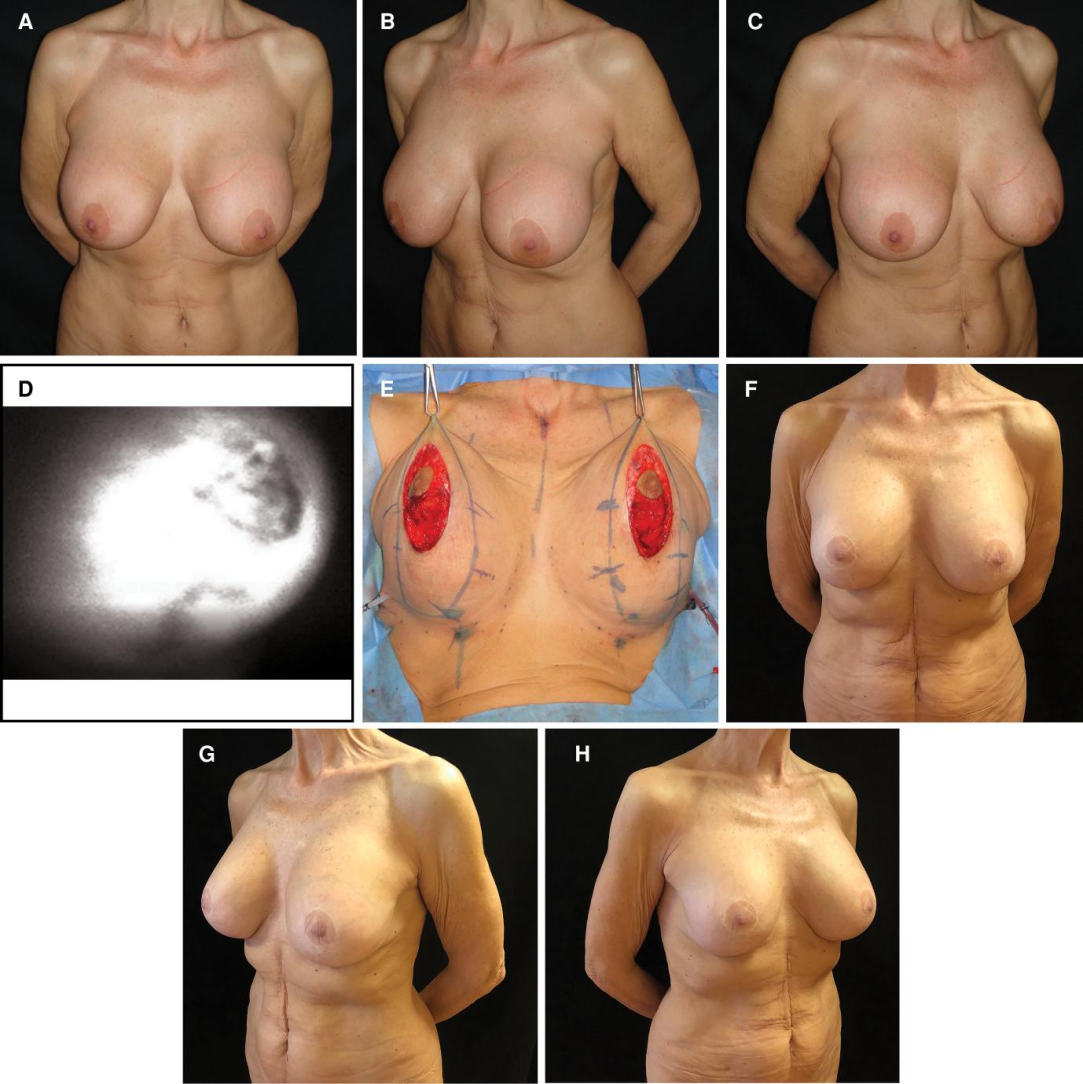
This 59-year-old female patient presented with breast shape deformity and Baker 3 capsular contracture after submuscular breast augmentation 20 years previously. Preoperative (A) frontal view and (B, C) lateral views. (D) After implant removal and superior capsulectomy through the periareolar incision of the previous surgery, the indocyanin green angiography detected normal perfusion. (E) Anatomical 310-cc implants were inserted in the submuscular pocket, and the J-scar mastopexy was performed. Postoperative (F) frontal and (G, H) views at 3-year follow-up.

Patients with wound dehiscence/necrosis were treated with minor debridement and advanced dressings without exposure or loss of the implant. The mean follow-up was 18 months, and the range was from 6 to 24 months. No complications were experienced in patients with submuscular implant placement (Figure 3).

## Discussion

ICG angiography to assess tissue vascularization is increasingly employed in plastic surgery^17^ and has various applications in oncologic breast surgery^18^ and implant-based and autologous breast reconstruction.^19^ ICG angiography is a minimally invasive, real-time imaging technique that allows a very precise evaluation of skin and soft-tissue perfusion.^20–23^ Swanson proposed the utilization of laser fluorescence imaging to evaluate the possibility of compromised circulation caused by inserting breast implants at the time of skin excision in augmentation mastopexy.^24^ Although secondary breast aesthetic surgery to resolve implant complications is commonly performed and the literature shows a relatively high complication rate,^4,5^ the utilization of ICG angiography in this context is not reported in the literature. Management of the residual breast tissue after implant removal can be challenging, especially if insertion of the new implant is required. The long-term presence of implants typically results in changes in breast anatomy and physiology, including parenchymal atrophy, tissue thinning, and reduction of breast envelope perfusion. Moreover, capsular contracture is common, and a total capsulectomy is widely recommended for its treatment.^7^ In a recent study,^25^ partial capsulectomy was reported as the most common modality for the treatment of capsular contracture (46.1% partial capsulectomy vs 35.1% total capsulectomy). Nevertheless, there is limited clinical evidence in in the literature behind the surgical management of capsular, and the data on capsulectomy are less conclusive.^26^ In our experience, after removing the implant, we prefer, if not strictly indicated (ie, calcified capsule, silicone-soaked capsule), not to remove the capsule in the lower quadrants in order not to further reduce their vascularization. In our study, total capsulectomy was required in 5 patients of the ICG angiography group, and a significant correlation was found between total capsulectomy and hypoperfusion. When planning secondary breast surgery, it would be desirable to consider the thickness of breast tissue overlying the implant and evaluate the blood supply to the NAC and the soft tissues of the inferior pole, because previous procedures may have compromised local perfusion.

Previous studies on the evaluation of tissue perfusion in secondary breast surgery were based on clinical evaluation. Handel suggested that if a surgeon is concerned about the viability of the NAC or mastopexy flaps during surgery, an implant should not be inserted; the patient may undergo a secondary procedure a few months later.^5^ Spring et al confirmed the clinical evaluation method and recommended minimizing tissue undermining and following previous incisions.^4^

In this study, we evaluated the utilization of ICG angiography to verify real-time, on the operating table, skin and mammary gland perfusion to aid decision-making during surgery. In our preliminary experience, the intraoperative evaluation with ICG in ICG angiography group allowed to identify a breast area with poor perfusion in 7 patients (44%). Having this information intraoperatively was fundamental because this made it possible to customize the surgery by removing the non-viable tissue, which will be responsible for the necrosis of the skin/soft tissues. According to our preliminary results with intraoperative ICG angiography, the most critical area was the central portion of the inferior pole in patients undergoing secondary breast surgery after subglandular breast augmentation. This is in agreement with the literature, which points out how—if the removed implant is large and subglandular—the overlying tissue can be stretched and thinned by the implant and further cosmetic surgery can compromise the residual blood supply, leading to necrosis of the gland or breast skin.^5^ From the comparison between the ICG angiography group and the non-ICG angiography group, we observed a higher complication rate in the non-ICG angiography group. We found a statistically significant association between the non-ICG angiography group and vertical-scar dehiscence or necrosis. Therefore, by employing this additional technology, complications related to the vertical scar would be avoided. No complications were experienced in patients with submuscular implant placement, confirming that the submuscular position of the implant represents a protective factor toward hypoperfusion-related complications.

Although conservative tissue undermining in secondary mastopexy decreases the possibility of impairing the tenuous blood supply, the risk of remodeling non-viable breast tissue is significant. Intraoperative ICG angiography can be crucial for the visualization of tissue with poor perfusion that needs to be removed. We believe that care should be taken in performing secondary surgery in these patients and that a real-time knowledge of breast perfusion can help to reduce complications due to hypoperfusion. The only drawback of adding this technology is the additional cost of intraoperative angiography, although the costs related to reoperations for any necrotic complications could justify the utilization of an undoubtedly expensive technology. Because a small number of patients were enrolled in this study, future studies based on a large population analysis could elucidate the factors that lead to changes in breast perfusion after breast augmentation and the incidence of this complication. With further studies with more patients, it may also be possible in the future to standardize the removal of poorly vascularized breast tissue in secondary breast surgery, taking into account factors such as implant volume, patient age, position of the implant, thickness of the skin, and the period since breast implantation. In lack of broader and clearer literature on this topic, in doubtful cases, we suggest the utilization of ICG angiography for safer surgical management of such cases.

## Conclusions

Safer secondary surgery can be offered to patients undergoing secondary cosmetic breast procedures by adding the intraoperative ICG angiography to surgical practice. When the details of the first augmentation surgery are unknown, for example, implant plane, type of pedicle employed, if the implant is large and subglandular, and if capsulectomy is performed, the evaluation of breast tissues perfusion with ICG angiography to identify the hypovascularized areas to remove them can be considered as a valid and useful method that can help the surgeon avoid complications such as skin and gland necrosis. However, in our preliminary study, the patient sample was too small, and the data consequently had low statistical power. Therefore, further studies are needed to confirm these results.

## References

[1] Aesthetic Plastic Surgery National Databank Statistics 2020–2021. Aesthet Surg J. 2022;42(Supplement_1):1–16. doi: 10.1093/asj/sjac116 35730469

[2] Araco A , Caruso R, Araco F, Overton J, Gravante G. Capsular contractures: a systematic review. Plast Reconstr Surg. 2009;124(6):1808–1819. doi: 10.1097/PRS.0b013e3181bf7f26 19952637

[3] Handel N , Cordray T, Gutierrez J, Jensen JA. A long-term study of outcomes, complications, and patient satisfaction with breast implants. Plast Reconstr Surg. 2006;117(3):757–767. doi: 10.1097/01.prs.0000201457.00772.1d 16525261

[4] Spring MA , Macias LH, Nadeau M, Stevens WG. Secondary augmentation-mastopexy: indications, preferred practices, and the treatment of complications. Aesthet Surg J. 2014;34(7):1018–1040. doi: 10.1177/1090820X14543943 25168806

[5] Handel N . Secondary mastopexy in the augmented patient: a recipe for disaster. Plast Reconstr Surg. 2006;118(7 Suppl):152S–163S; discussion 164S-165S, 166S-167S. doi: 10.1097/01.prs.0000246106.85435.74 17099496

[6] Teng E , Broer N, Heidekrueger P, et al In vivo changes of breast perfusion after augmentation. Aesthet Surg J. 2016;36(10):1133–1140. doi: 10.1093/asj/sjw139 27625032

[7] Calobrace MB , Mays C. An algorithm for the management of explantation surgery. Clin Plast Surg. 2021;48(1):1–16. doi: 10.1016/j.cps.2020.09.005 33220896

[8] Clemens MW , Medeiros LJ, Butler CE, et al Complete surgical excision is essential for the management of patients with breast implant-associated anaplastic large-cell lymphoma. J Clin Oncol. 2016;34(2):160–168. doi: 10.1200/JCO.2015.63.3412 26628470 PMC4872006

[9] Spear SL , Baker JL Jr. Classification of capsular contracture after prosthetic breast reconstruction. Plast Reconstr Surg. 1995;96(5):1119–1123. discussion 1124. doi: 10.1097/00006534-199510000-00018 7568488

[10] Phillips BT , Lanier ST, Conkling N, et al Intraoperative perfusion techniques can accurately predict mastectomy skin flap necrosis in breast reconstruction: results of a prospective trial. Plast Reconstr Surg. 2021;129(5):778e–788e. doi: 10.1097/PRS.0b013e31824a2ae8 22544108

[11] Gasperoni C , Salgarello M, Gasperoni P. A personal technique: mammaplasty with J scar. Ann Plast Surg. 2002;48(2):124–130. doi: 10.1097/00000637-200202000-00002 11910216

[12] Salgarello M , Visconti G. Short-scar augmentation mastopexy in massive-weight loss patients: four-step surgical principles for reliable and reproducible results. Aesthetic Plast Surg. 2020;44(2):272–282. doi: 10.1007/s00266-019-01540-0 31797044

[13] Hall-Findlay EJ . A simplified vertical reduction mammaplasty: shortening the learning curve. Plast Reconstr Surg. 1999;104(3):748–759; discussion 760-763. doi: 10.1097/00006534-199909010-00020 10456528

[14] Lejour M , Abboud M. Vertical mammaplasty without inframammary scar and with breast liposuction. Perspect Plast Surg. 1990;4:67–90. doi: 10.1055/S-2008-1080455

[15] Salgarello M , Visconti G, Barone-Adesi L. Interlocking circumareolar suture with undyed polyamide thread: a personal experience. Aesthetic Plast Surg. 2013;37(5):1061–1062. doi: 10.1007/s00266-013-0186-1 23860820

[16] Hammond DC , Khuthaila DK, Kim J. The interlocking Gore-Tex suture for control of areolar diameter and shape. Plast Reconstr Surg. 2007;119(3):804–809. doi: 10.1097/01.prs.0000251998.50345.e9 17312481

[17] Burnier P , Niddam J, Bosc R, Hersant B, Meningaud JP. Indocyanine green applications in plastic surgery: a review of the literature. J Plast Reconstr Aesthet Surg. 2017;70(6):814–827. doi: 10.1016/j.bjps.2017.01.020 28292569

[18] Struk S , Honart J-F, Qassemyar Q, et al Use of indocyanine green angiography in oncological and reconstructive breast surgery. Ann Chir Plast Esthet. 2018;63(1):54–61. doi: 10.1016/j.anplas.2017.09.008 29107433

[19] Griffiths M , Chae MP, Rozen WM. Indocyanine green-based fluorescent angiography in breast reconstruction. Gland Surg. 2016;5(2):133–149. doi: 10.3978/j.issn.2227-684X.2016.02.01 27047782 PMC4791345

[20] Reinhart MB , Huntington CR, Blair LJ, Heniford BT, Augenstein VA. Indocyanine green: historical context, current applications, and future considerations. Surg Innov. 2016;23(2):166–175. doi: 10.1177/1553350615604053 26359355

[21] Damsgaard TE , Rønning H. Indocyanine green guided mastectomy and immediate breast reconstruction. Gland Surg. 2019;8(Suppl 4):S287–S290. doi: 10.21037/gs.2019.06.10 31709169 PMC6819884

[22] Silva Neto E , Figueiredo PHM, Moro MG, et al Use of laser-assisted indocyanine green angiography in breast reconstruction: systematic review and meta-analysis. J Surg Oncol. 2020;121(5):759–765. doi: 10.1002/jso.25782 31773735

[23] Desmettre T , Devoisselle JM, Soulie-Begu S, Mordon S. Fluorescence properties and metabolic features of indocyanine green (ICG). J Fr Ophtalmol. 1999;22(9):1003-1016. 10609179

[24] Swanson E . Safety of vertical augmentation-mastopexy: prospective evaluation of breast perfusion using laser fluorescence imaging. Aesthet Surg J. 2015;35(8):938–949. doi: 10.1093/asj/sjv086 26508647

[25] Hidalgo DA , Sinno S. Current trends and controversies in breast augmentation. Plast Reconstr Surg. 2016;137(4):1142–1150. doi: 10.1097/01.prs.0000481110.31939.e4 27018669

[26] Wan D , Rohrich RJ. Revisiting the management of capsular contracture in breast augmentation: a systematic review. Plast Reconstr Surg. 2016;137(3):826–841. doi: 10.1097/01.prs.0000480095.23356.ae 26910663

